# Supplementary figures and images for: TMED9 coordinates the clearance of misfolded GPI-anchored proteins out of the ER and into the Golgi
Source: PLoS Biol. 2025 Apr 9;23(4):e3003084. doi: 10.1371/journal.pbio.3003084 (PMC12052135; doi:10.1371/journal.pbio.3003084)

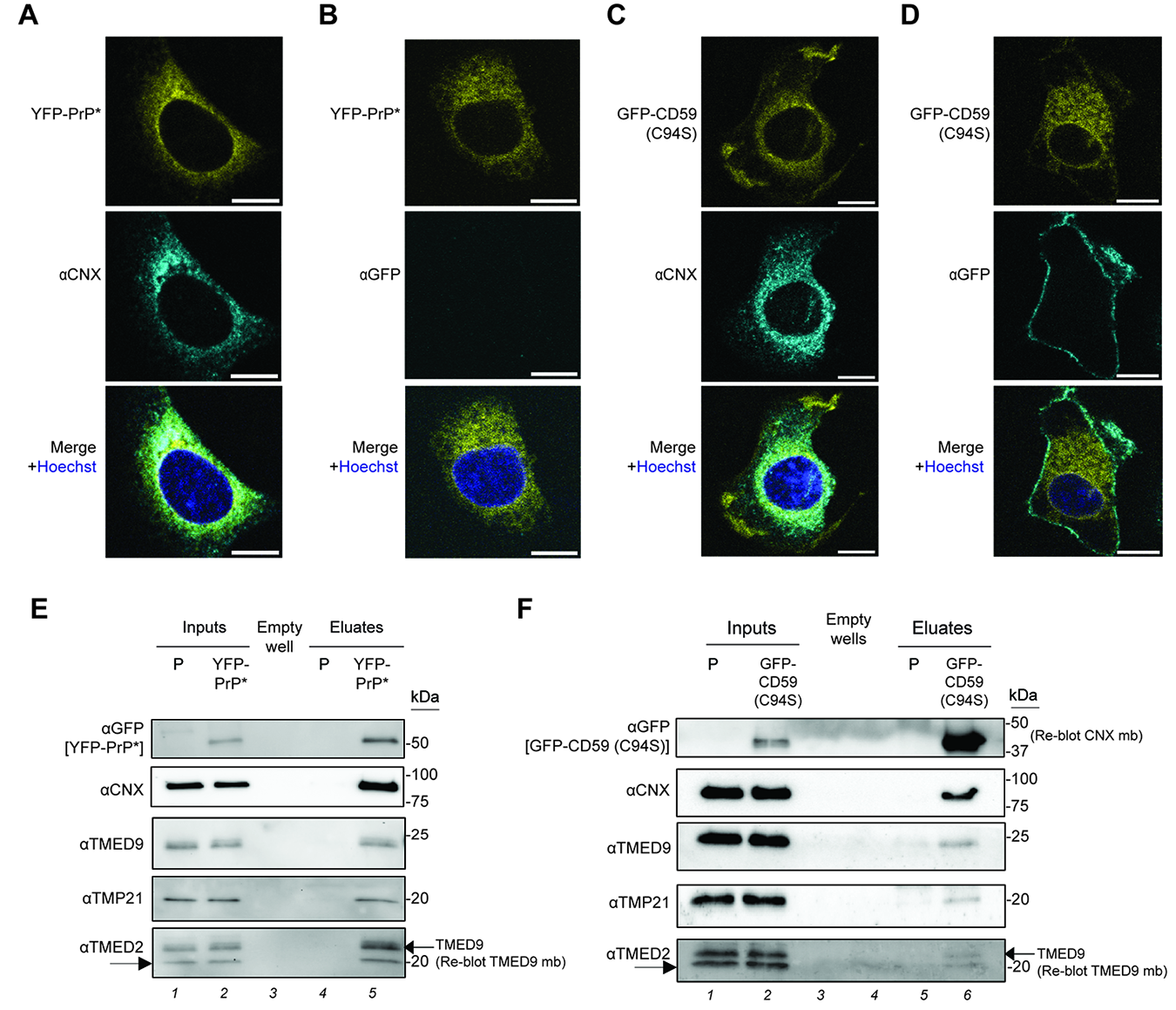

Supplement: S1 Fig — (A and B) Confocal images of YFP-PrP * NRK cells at steady state. Scale bar represents 10 µm. (A) Immunofluorescence image of endogenous calnexin (CNX) in a YFP-PrP * NRK cell. The nucleus was stained with Hoechst. (B) Immunofluorescence image of cell-surface YFP-PrP * using anti-GFP antibody on cells that were not permeabilized. The nucleus was stained with Hoechst. (C and D) Confocal images of GFP-CD59 (C94S) NRK cells at steady state. Scale bar represents 10 µm. (C) Immunofluorescence image against endogenous calnexin (CNX) in GFP-CD59 (C94S). The nucleus was stained with Hoechst. (D) Immunofluorescence of cell-surface GFP-CD59 (C94S) using anti-GFP antibody on cells that were not permeabilized. The nucleus was stained with Hoechst. (E) Western blots of GFP-tag co-immunoprecipitates from the parental untransfected NRK cells (P) or stably transfected YFP-PrP * NRK cells at steady state (n = 1). Blots were probed for GFP for YFP-PrP * , and probed for endogenous calnexin (CNX), TMP21, TMED2, and TMED9. (F) Western blots of GFP-tag co-immunoprecipitates from parental untransfected NRK cells (P) or stably transfected GFP-CD59 (C94S) NRK cells (n = 1). Blots were probed for GFP for GFP-CD59 (C94S) and probed for endogenous calnexin (CNX), TMP21, TMED2, and TMED9. (TIF) [file pbio.3003084.s001.tif]

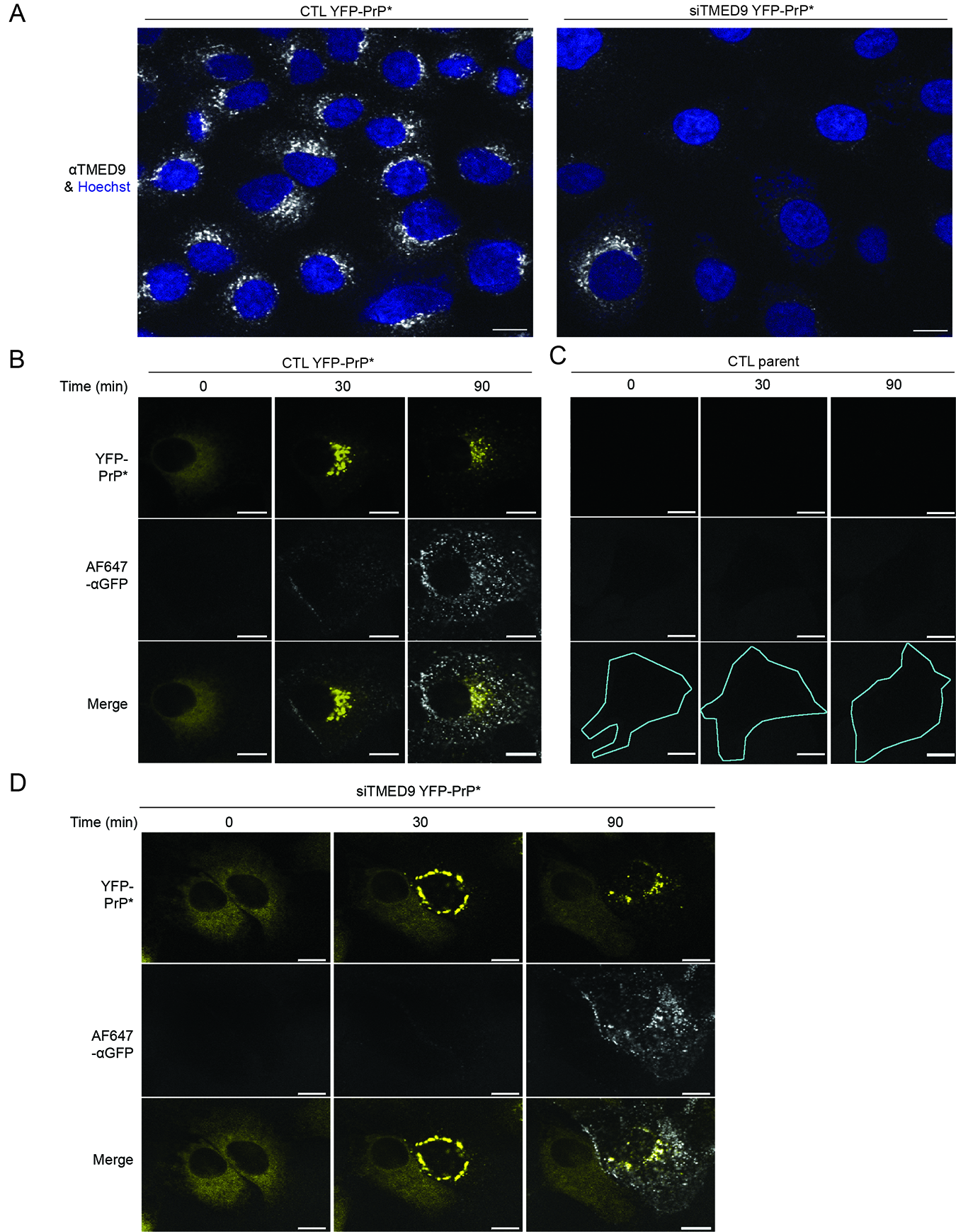

Supplement: S2 Fig — (A) Immunofluorescence images of TMED9 staining in YFP-PrP * NRK cells that were treated with scrambled control siRNA (CTL) or siRNA against TMED9 (siTMED9). With our TMED9-knockdown protocol, the majority of siTMED9-treated YFP-PrP * NRK cells demonstrated TMED9 knock-down. A minor population (~30%–35%) of the siTMED9-treated cells did not demonstrate knock-down of TMED9. (B–D) Time-lapse images of cells collected immediately after the addition of TG, Alexa Fluor 647 (AF647)-conjugated rabbit anti-GFP antibodies, and bafilomycin A1. There is a faint ambient signal from the AF647-conjugated antibody in the medium that can be enhanced by increasing the gain. Scale bar represents 10 µm. (B) YFP-PrP * NRK cell that was treated with scrambled control siRNA (CTL). (C) Parental untransfected NRK cell that was treated with scrambled control siRNA (CTL). The outline of the cell has been hand-traced in cyan. (D) YFP-PrP * NRK cells that were treated with siRNA against TMED9 (siTMED9). A field of view that contained one cell that displayed the phenotype of complete TMED9 knockdown versus one cell with incomplete knockdown was selected for comparison. This panel is associated with S2 Video. (TIF) [file pbio.3003084.s002.tif]

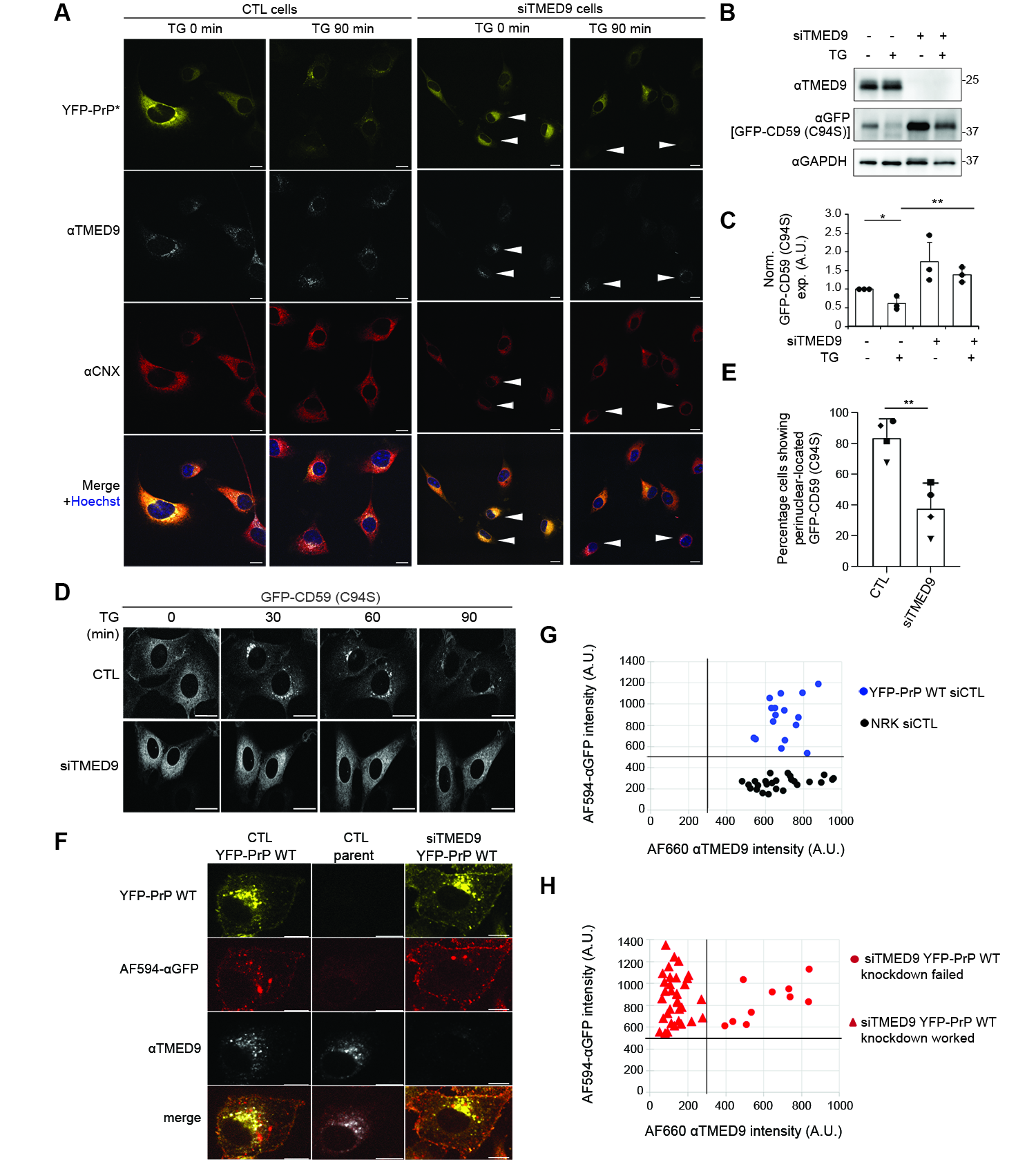

Supplement: S3 Fig — (A) Immunofluorescence image stained for endogenous TMED9 and CNX in control YFP-PrP * NRK (CTL) or siTMED9-treated YFP-PrP * NRK cells (siTMED9 cells) at steady state (TG 0 min) after 90 min of TG-treatment (TG 90 min). Nuclei were stained with Hoechst. For the siTMED9-treated cells, large white arrows point to cells with only partial TMED9 knockdown. Scale bar represents 10 µm. (B) Representative western blots of either control (CTL) or TMED9 siRNA (siTMED9)-treated GFP-CD59 (C94S) NRK cells that were either untreated (TG−) or treated with 90 min TG (TG+ ) (n = 3 biological replicates). (C) Bar graph representing the mean band intensity for GFP-CD59 (C94S) from three biological replicates as shown in (B). For each condition, GFP-CD59 (C94S) band intensities were double normalized. First, GFP band intensity was normalized against the band intensity of GAPDH. Second, the GFP band intensity was normalized against the untreated control (“siTMED9− TG−”) band intensity. Error bars represent standard deviation of the mean. Symbols were coded for each independently performed experiment. Statistics were calculated from unpaired t test with Welch’s correction with * indicated p < 0.05 and ** indicated p < 0.01. (D) Time-lapse images of control (CTL) or TMED9 siRNA (siTMED9) GFP-CD59 (C94S) NRK cells. Image-collection was started immediately after the addition of TG. Scale bar represents 20 µm. (E) Percentage of cells showing a perinuclear Golgi-pattern for GFP-CD59 (C94S), indicative of Golgi-localization, after 30 min of TG-treatment. These data are derived from four independent experiments (n = 4). Symbols were coded for each independently performed experiment. The number of cells analyzed for each biological replicate are as follows (CTL: triangle 37, diamond 59, circle 18, square 54; siTMED9: triangle 73, diamond 93, circle 28, square 64). Error bars represent standard deviation of the mean. Statistics were calculated from unpaired t test with Welch’s correction with ** [file pbio.3003084.s003.tif]

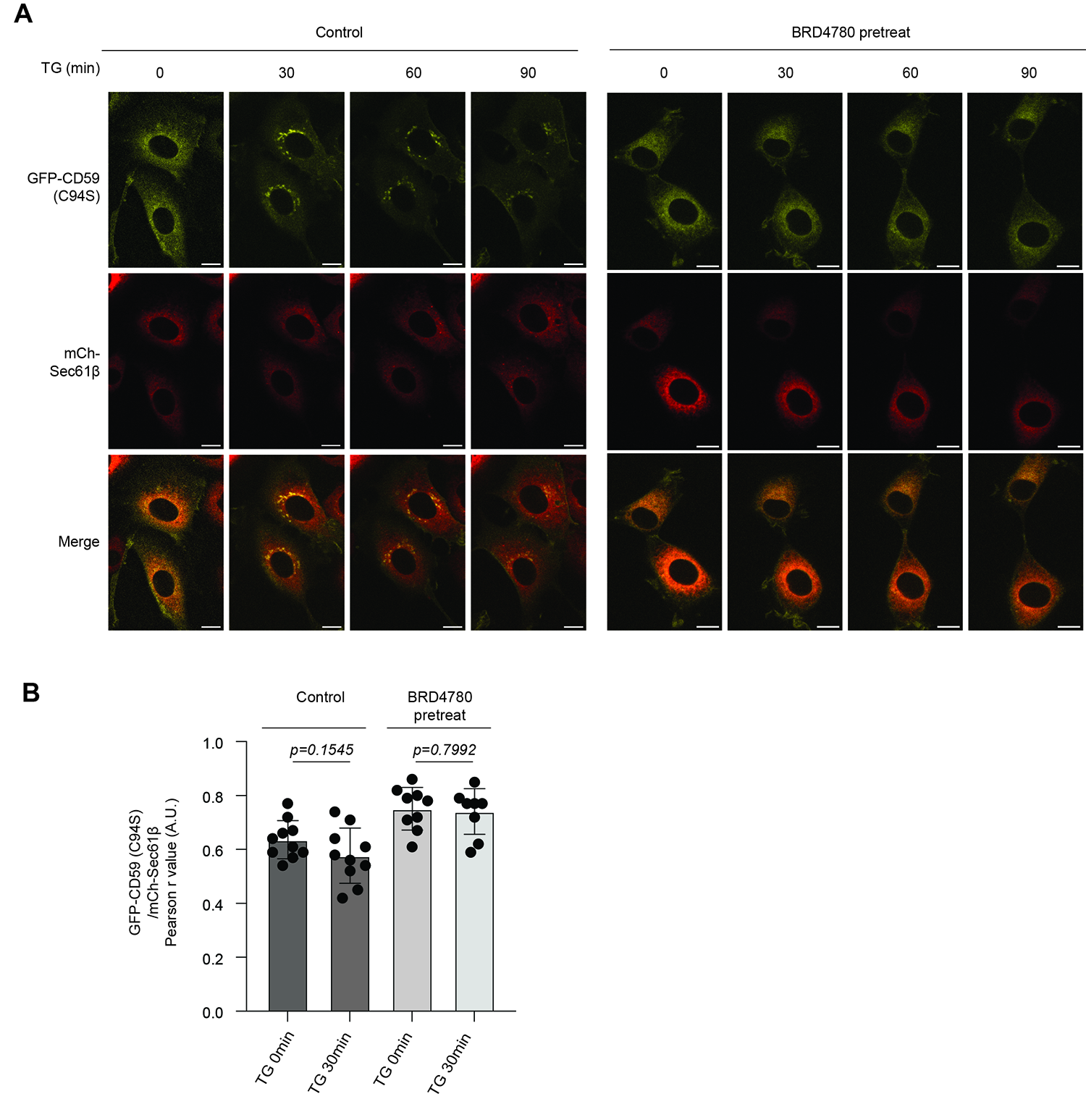

Supplement: S4 Fig — (A) Representative time-lapse imaging of stably transfected GFP-CD59 C94S NRK cells in control condition (CTL) or pretreated with BRD4780 for 30 min (pretreated with BRD4780). Image-collection was started immediately after the addition of TG. Scale bars represent 10 µm. (B) Plot of the average Pearson’s r values between GFP-CD59 (C94S) and mCh-Sec61β for Control versus BRD4780-pretreated conditions, as described in (A). For Control and BRD4780-pretreated conditions, 10 and 9 time-lapses of individual cells, respectively, were analyzed for the 0 and 30 min time points. Pearson’s colocalization coefficients, r, were measured between GFP-CD59 (C94S) and mCh-Sec61β within the boundaries of the cell. For each data point, the boundaries of the cells were revealed by temporarily maximizing the gain for mCh-Sec61β. In CTL cells, the r values between GFP-CD59 (C94S) and mCh-Sec61β for 0 and 30 min time points were 0.636 ± 0.067 and 0.577 ± 0.065, respectively. In BRD4780-treated cells, the r values between GFP-CD59 (C94S) and mCh-Sec61β for for 0 and 30 min time points were 0.751 ± 0.075 and 0.741 ± 0.080, respectively. The data underlying the graphs shown in S4 Fig are included in the S1 Data file. (TIF) [file pbio.3003084.s004.tif]

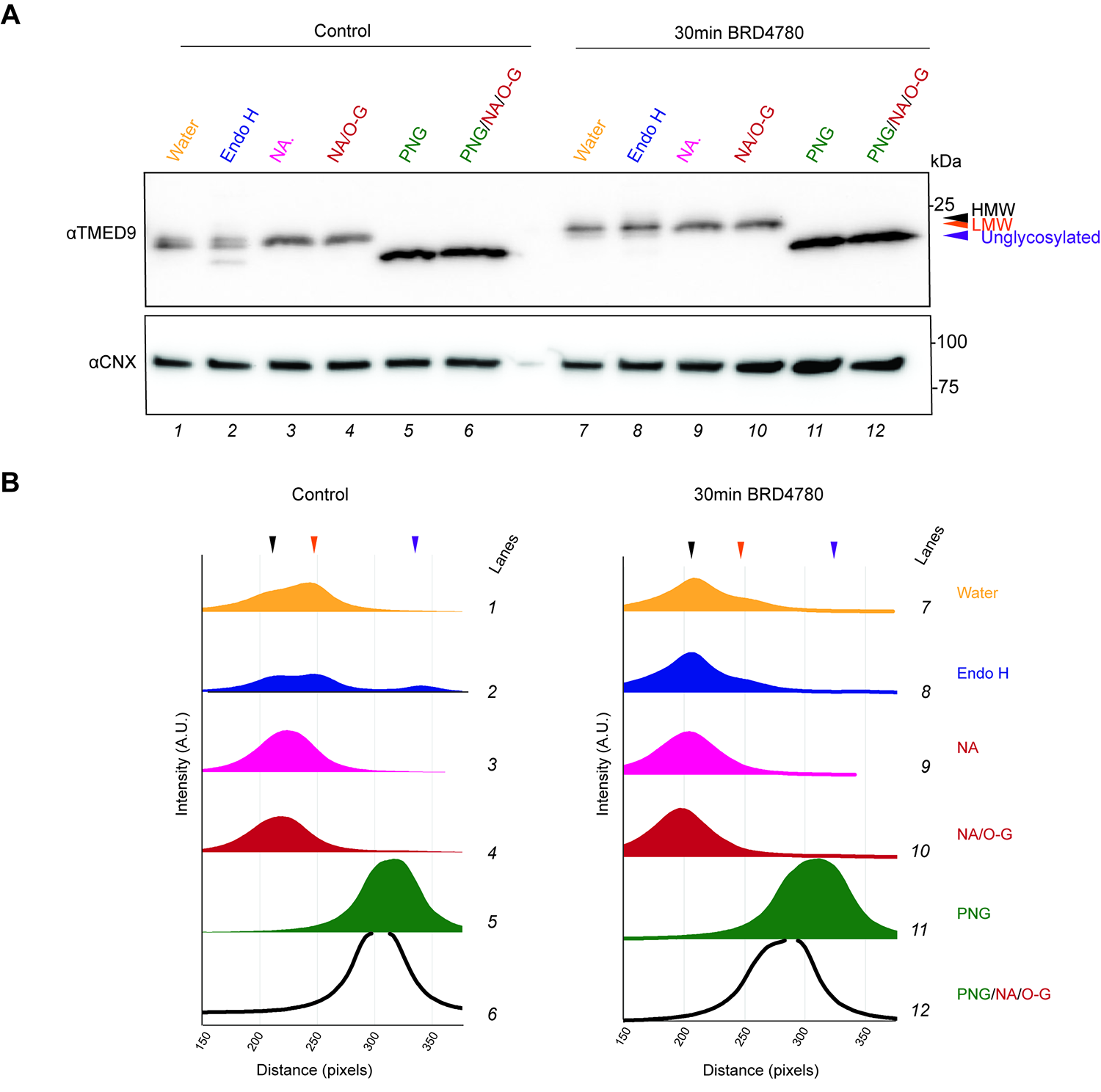

Supplement: S5 Fig — (A) Western blots of digested lysates of YFP-PrP * NRK cells in control and BRD4780-treated conditions. Protein lysates were not digested (water) or digested with Endoglycosidase H (Endo H), Neuraminidase only (NA), Neuraminidase + O-Glycosidase (NA + O-G.), Peptide-N-glycosidase F (PNG), or Neuraminidase + O-Glycosidase + Peptide-N-glycosidase F (NA + O-G + PNG) for 3 h at 37oC (n = 1 experiment). Blots were probed for TMED9 and calnexin (CNX). (B) western blot band graphics were obtained by first analyzing the plot lanes and second by generating line graphs of pre-selected ROIs from the western blot for control samples (left) or BRD4780 (30 min)-treated samples (right). Black arrow represents the HMW form, orange arrow represents the LMW and purple, the unglycosylated form of TMED9. The data underlying the graphs shown in S5 Fig are included in the S1 Data file. (TIF) [file pbio.3003084.s005.tif]

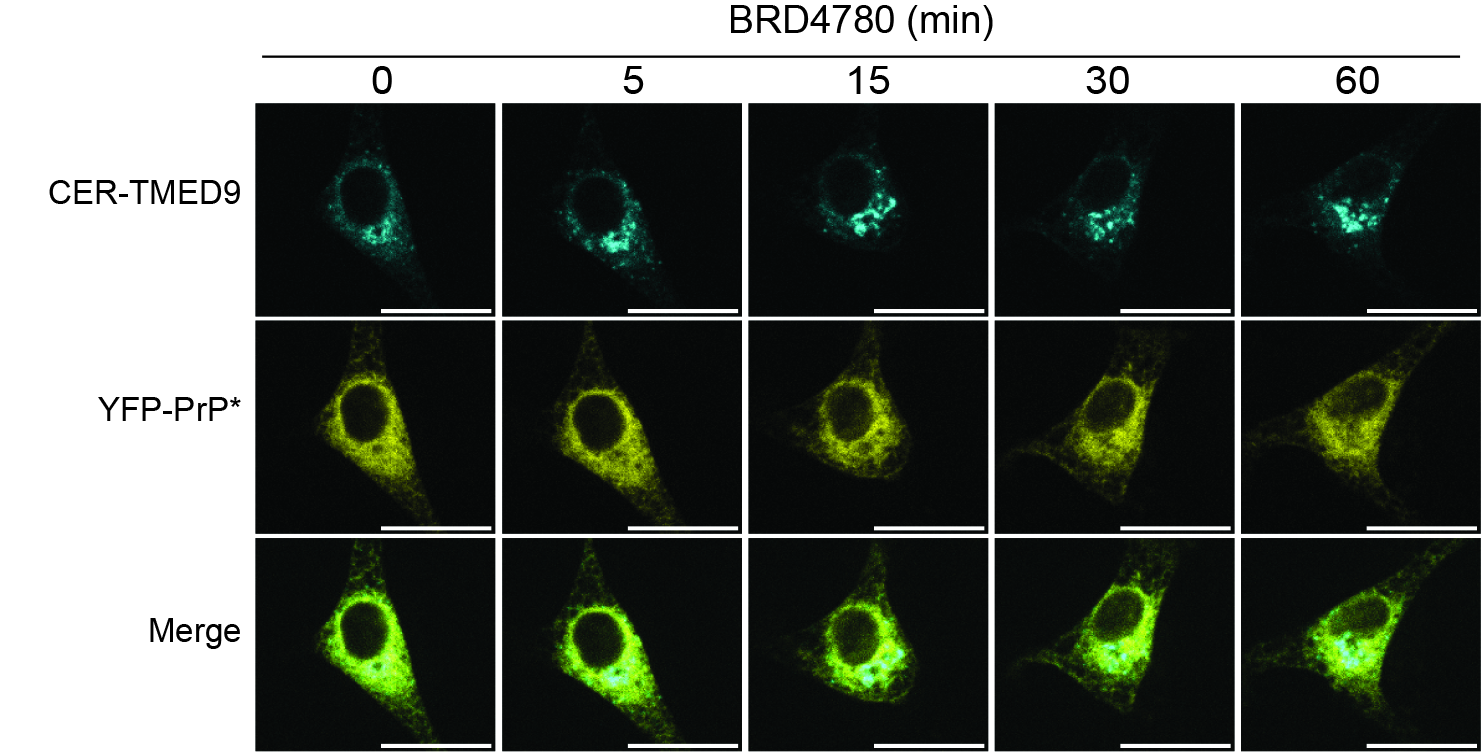

Supplement: S6 Fig — Time-lapse images of a typical YFP-PrP * NRK cell that was transfected with CER-TMED9. Time-lapse image collection was started immediately after the addition of 100 µM BRD4780 treatment. Scale bar represents 20 µm. (TIF) [file pbio.3003084.s006.tif]

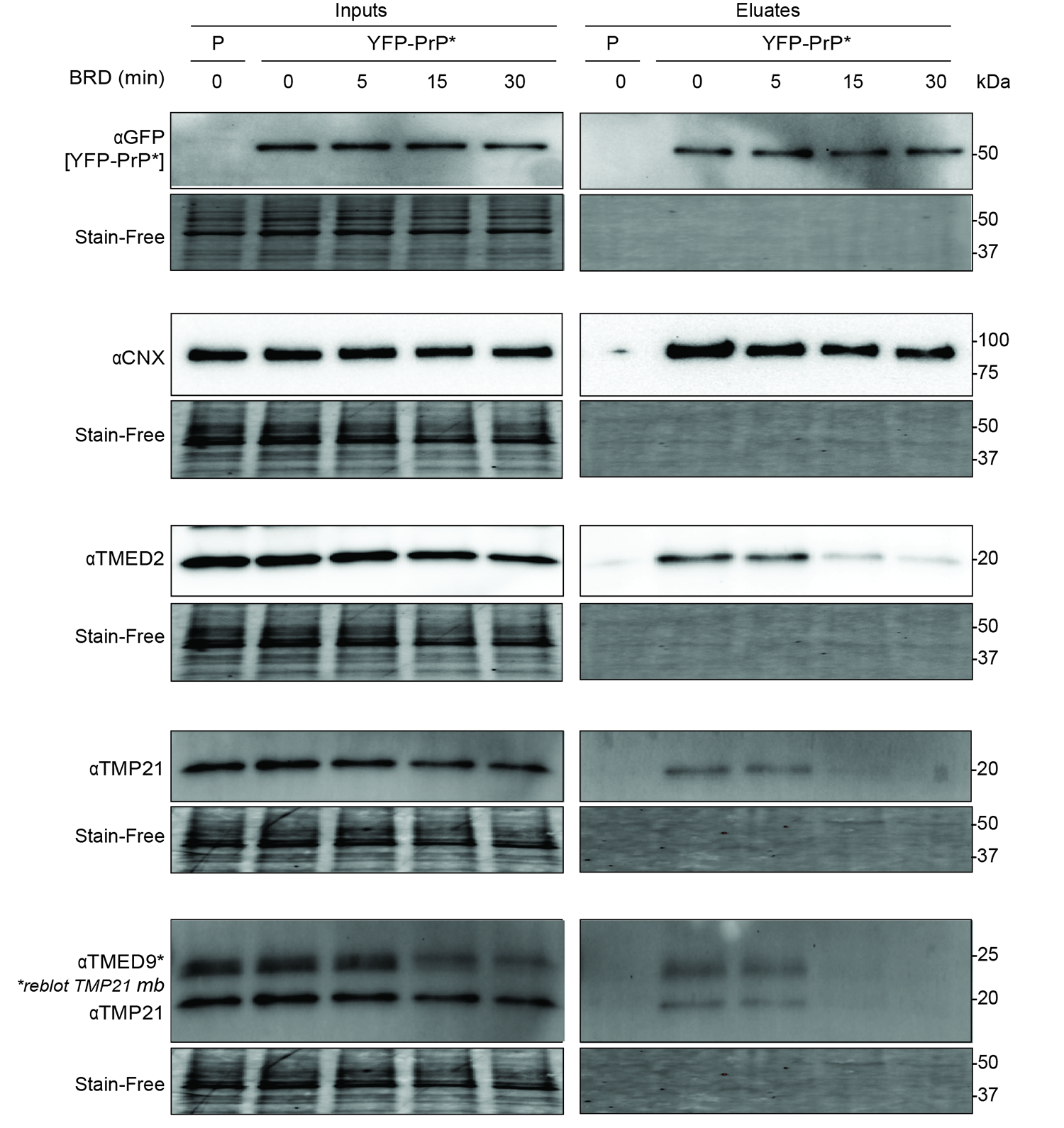

Supplement: S7 Fig — Western blots of GFP-tag co-immunoprecipitations (co-IPs) from the parental untransfected NRK cells (P) or stably transfected YFP-PrP * NRK cells (n = 1 experiment). YFP-PrP * NRK cells were treated with 100 µM BRD4780 (BRD) and collected for co-IP at the indicated time points. Cells were harvested at the indicated time points for co-immunoprecipitation of YFP-PrP * with anti-GFP antibody conjugated beads in addition to GFP to detect co-immunoprecipitation of YFP-PrP * constructs, and blots were probed for endogenous calnexin (CNX), TMED2, TMP21, and TMED9. Under each western blot is depicted a “Stain-Free” image of the total protein in the gel. (TIF) [file pbio.3003084.s007.tif]

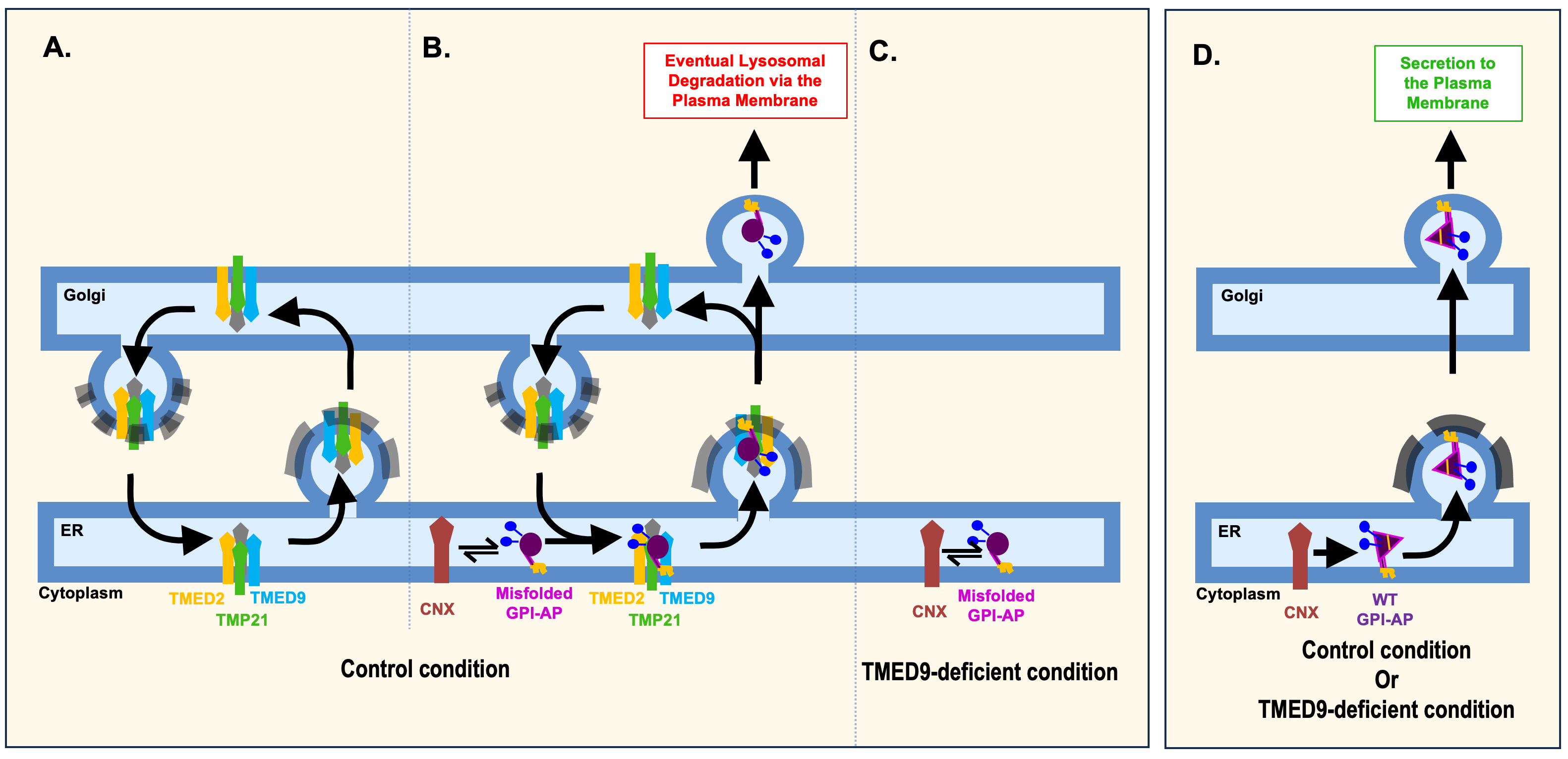

Supplement: S8 Fig — (A) At steady-state, TMED9 (cyan), in conjunction with TMP21 (green), TMED2 (gold), and likely other export machinery (gray), such as other p24-family members, cargo receptors, COPII and COPI coat proteins (gray), and traffic between the ER and Golgi, creating a pre-existing pathway for the ER-to-Golgi transport of select substrates. (B) Upon release from calnexin (red) during steady-state or ER-stress conditions, misfolded GPI-anchored proteins (GPI-APs) (depicted as purple circles) piggyback with p24-family members to access these p24-family populated ER-exit sites. (C) When TMED9 is depleted from the ER either by siRNA knockdown or by BRD4780-treatment, the TMED9 and p24-family populated ER-exit pathway collapses and misfolded GPI-APs are unable to exit the ER to the Golgi. Instead, they remain in association with calnexin. (D) By contrast to misfolded GPI-APs, properly folded GPI-APs are able to exit the ER to the Golgi for subsequent secretion to the plasma membrane regardless of the presence or absence of TMED9. Properly folded GPI-APs are depicted as purple triangles with a yellow stripe to indicate intact disulfide bonds. The p24-family members are not depicted in this panel. (PNG) [file pbio.3003084.s008.png]
