# Supplementary material for: TMED9 coordinates the clearance of misfolded GPI-anchored proteins out of the ER and into the Golgi
Source: PLoS Biol. 2025 Apr 9;23(4):e3003084. doi: 10.1371/journal.pbio.3003084 (PMC12052135; doi:10.1371/journal.pbio.3003084)

Ronzier and Satpute-Krishnan
Supplementary Blot Raw Data

Figure 2

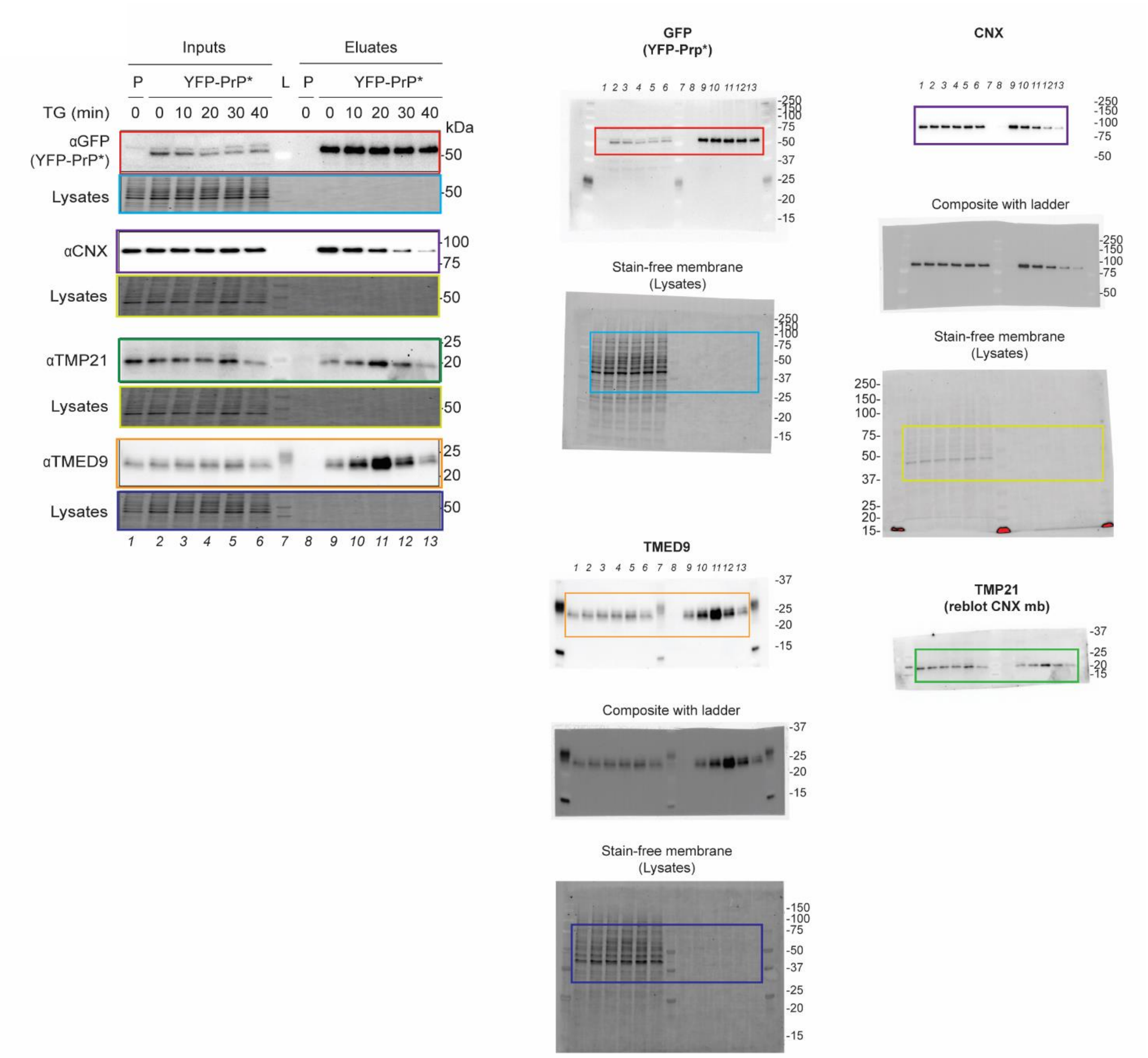

Ronzier and Satpute-Krishnan
Supplementary Blot Raw Data

Figure 3

Fig 3A

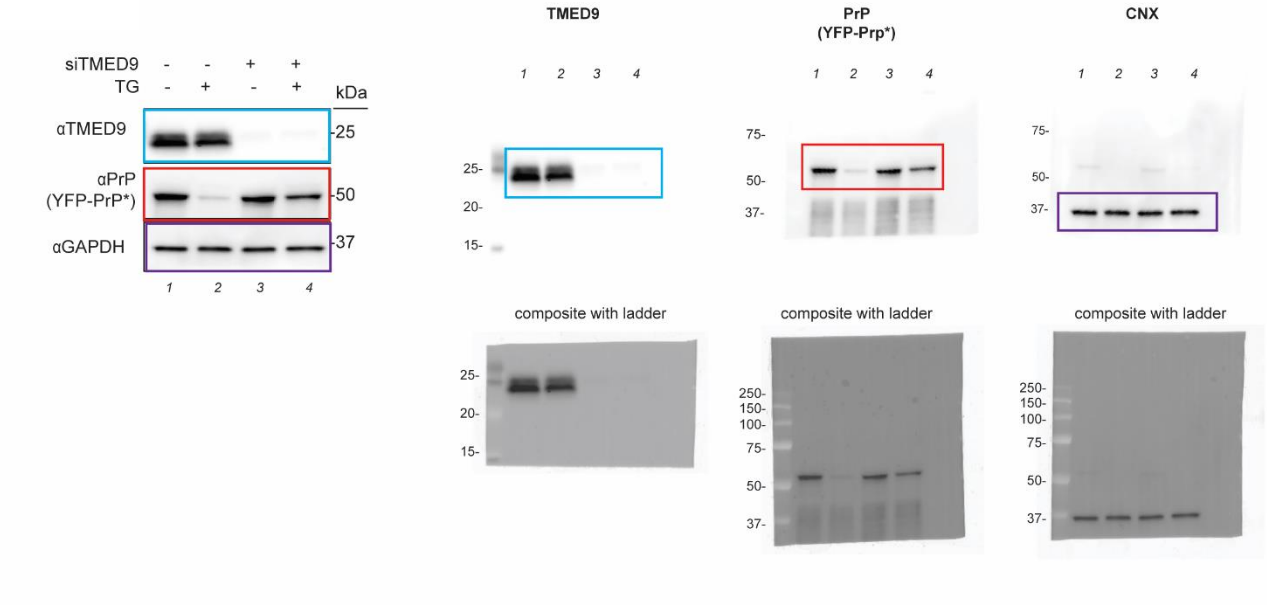

Ronzier and Satpute-Krishnan
Supplementary Blot Raw Data

Figure 4

Fig 4A

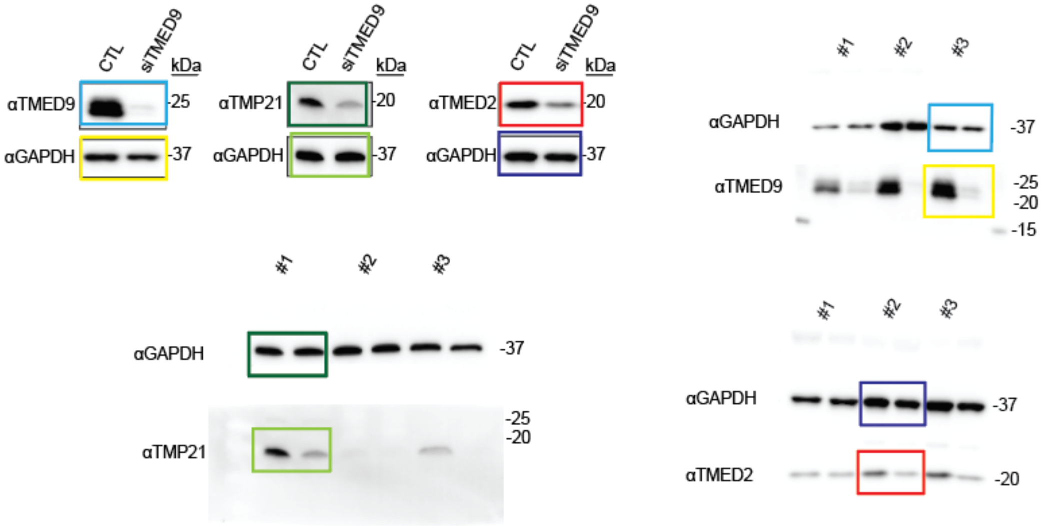

Fig 4E

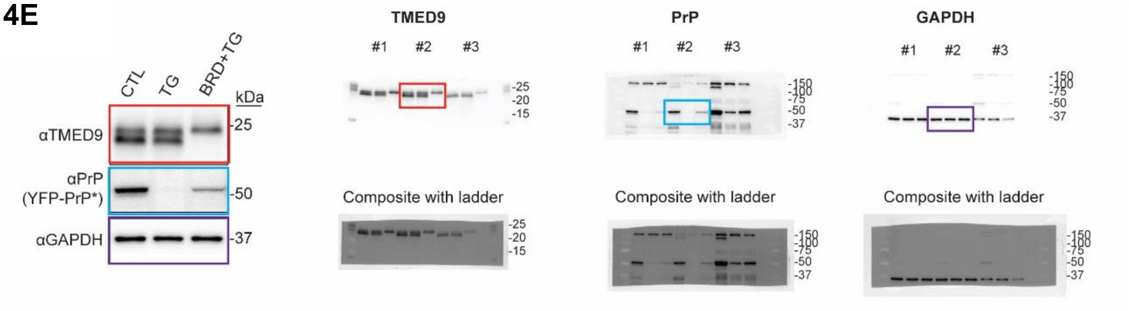

Fig 4G

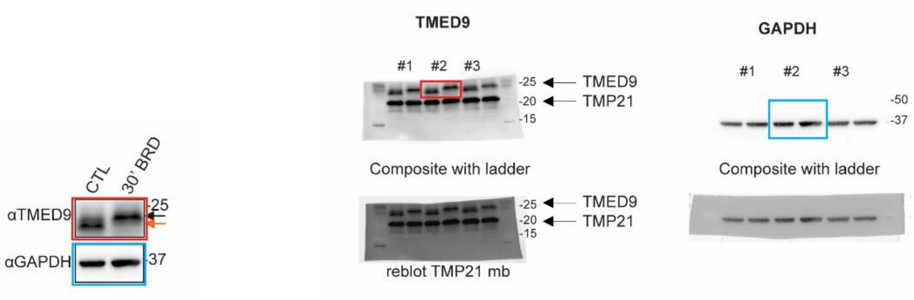

Ronzier and Satpute-Krishnan
Supplementary Blot Raw Data

Fig 4I

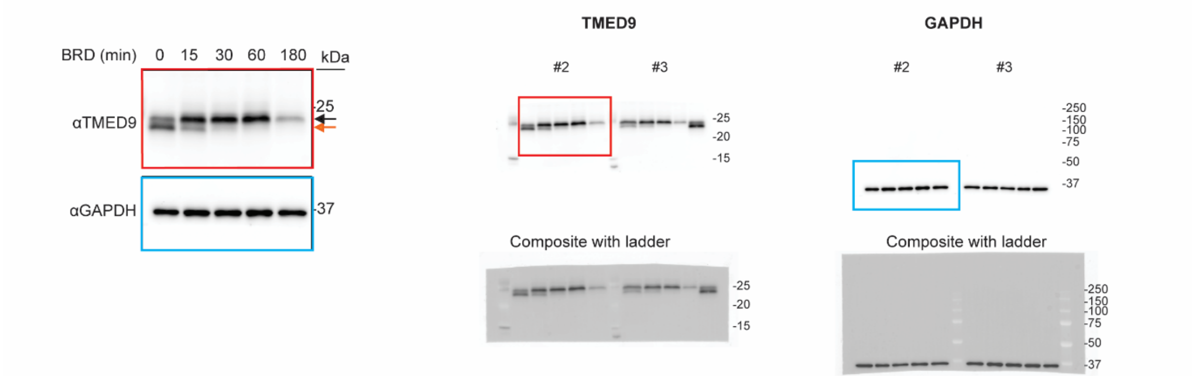

Fig 4K

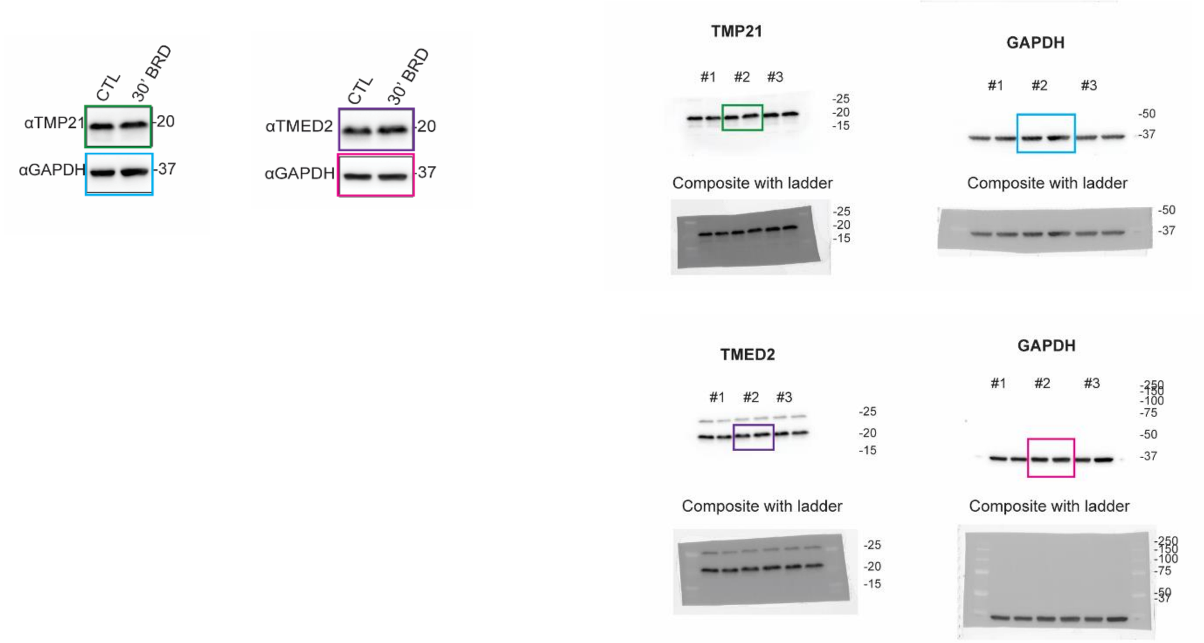

Ronzier and Satpute-Krishnan
Supplementary Blot Raw Data

Figure 5

Fig 5A

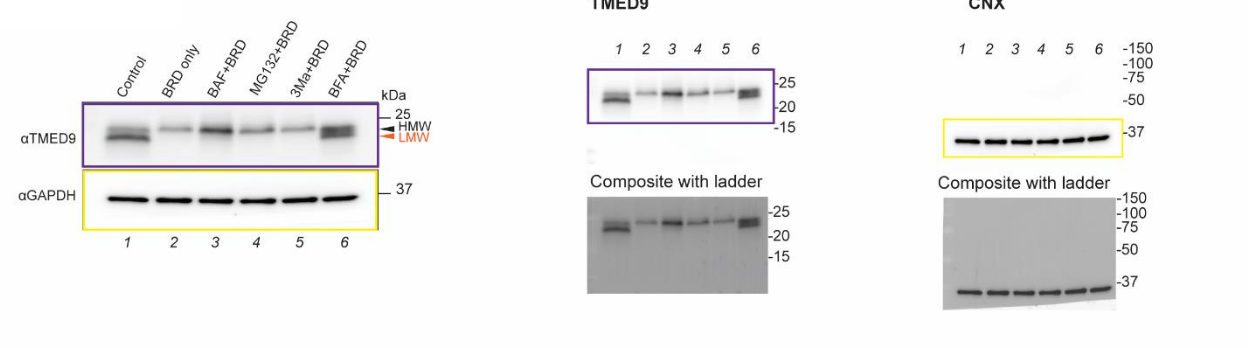

Fig 5C

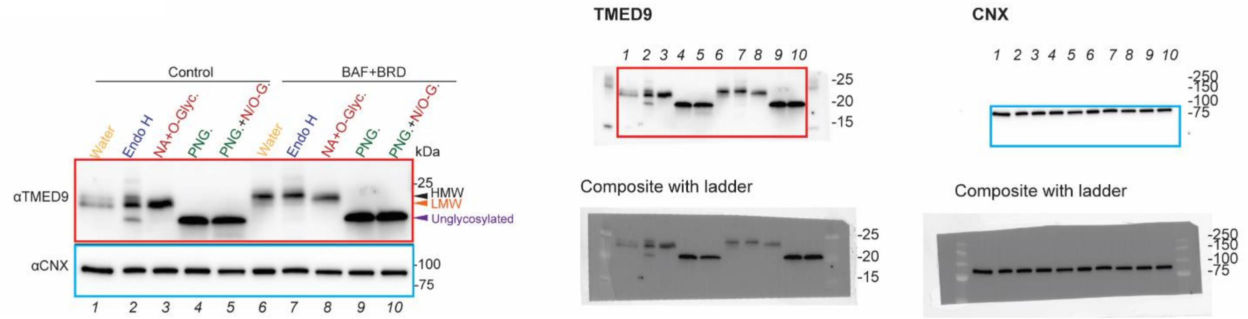

Figure 6

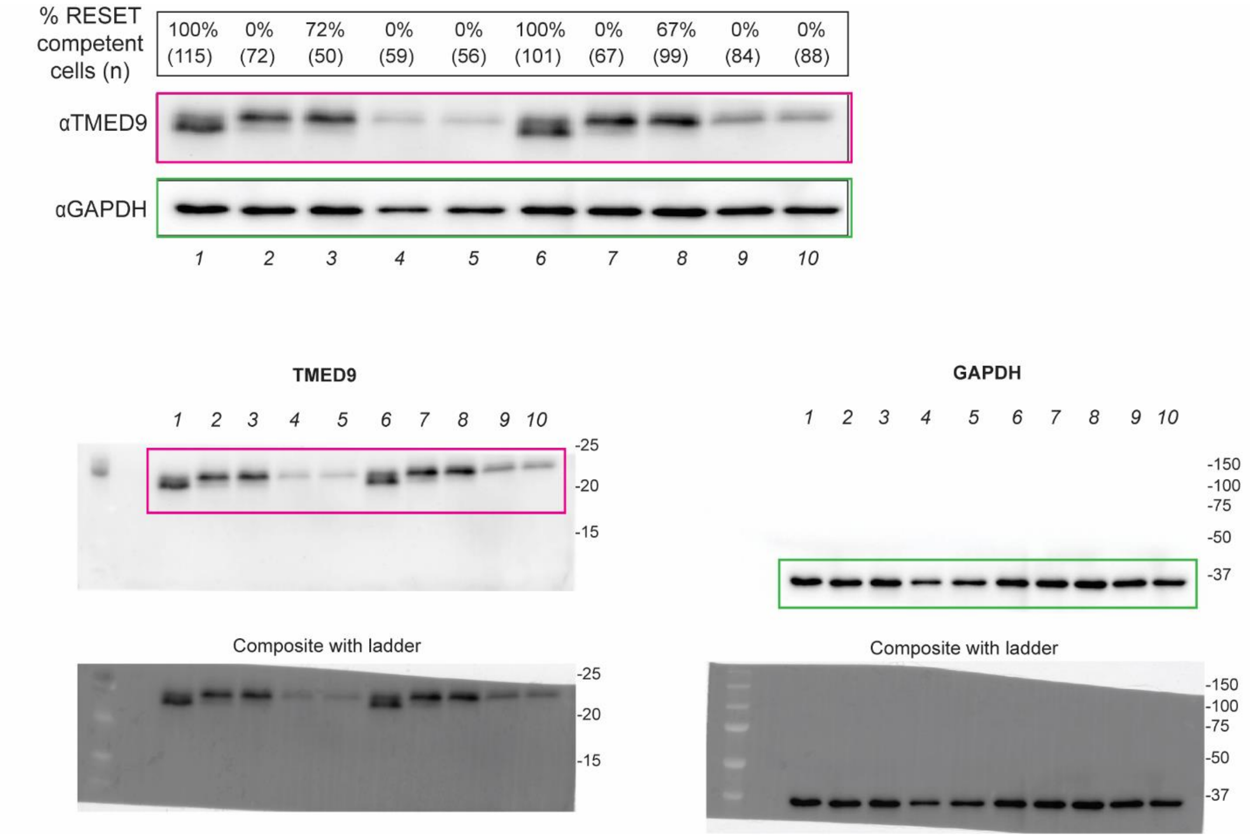
% RESET competent cells (n)
100% (115)
0% (72)
72% (50)
0% (59)
0% (56)
100% (101)
0% (67)
67% (99)
0% (84)
0% (88)
αTMED9
αGAPDH
1 2 3 4 5 6 7 8 9 10
TMED9
GAPDH
-25
-20
-15
-150
-100
-75
-50
-37
Composite with ladder
Composite with ladder

Ronzier and Satpute-Krishnan
Supplementary Blot Raw Data

Figure 7

Fig 7B

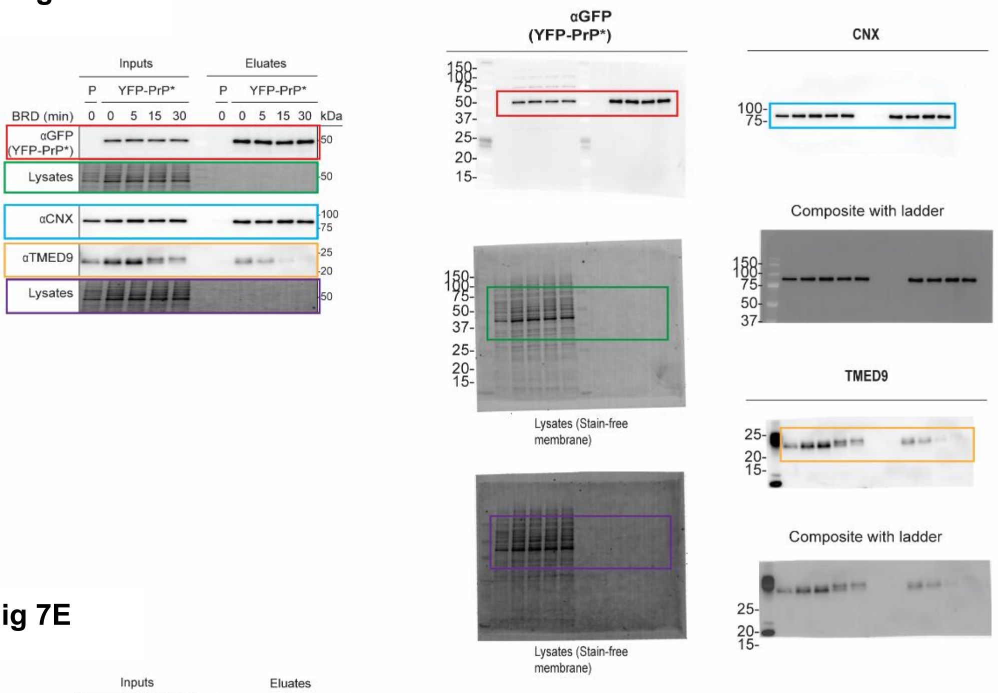

Fig 7E

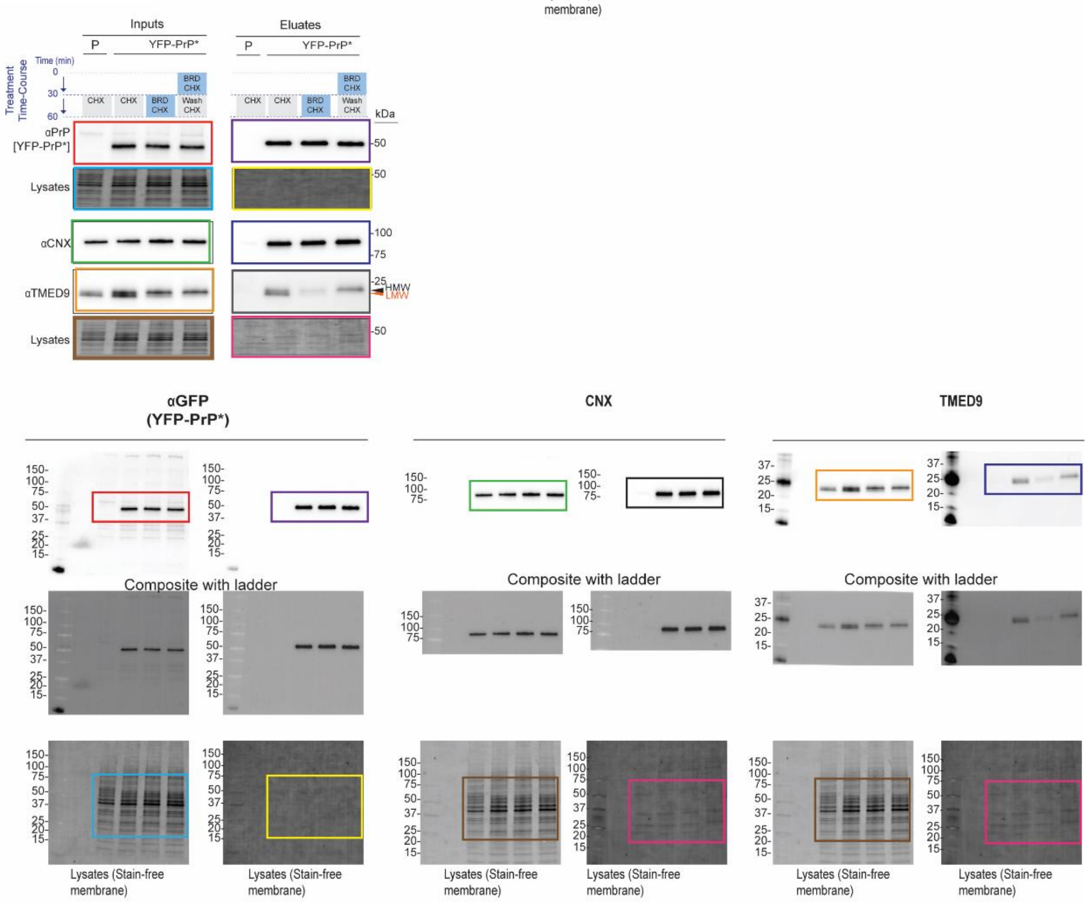

Ronzier and Satpute-Krishnan
Supplementary Blot Raw Data

Figure S1

Fig S1E

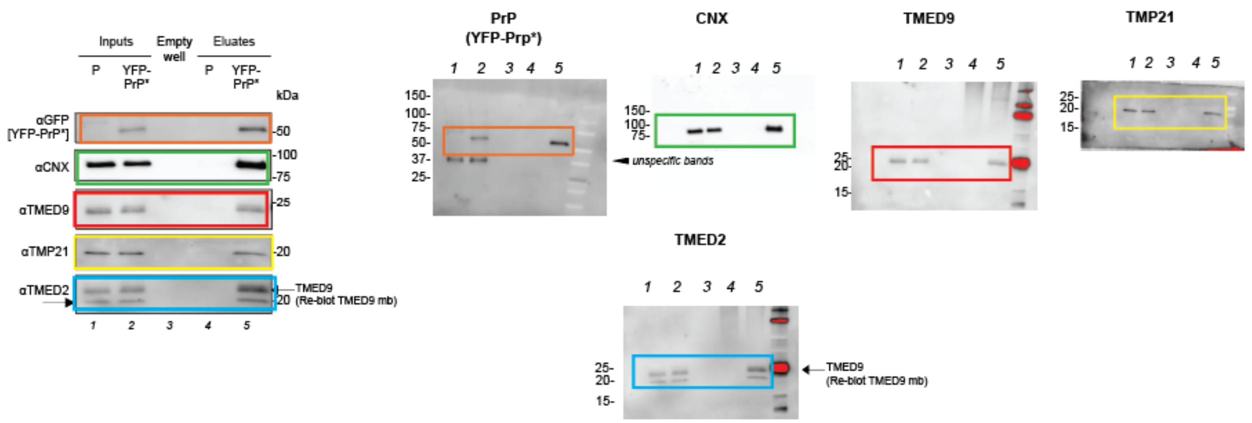

Fig S1F

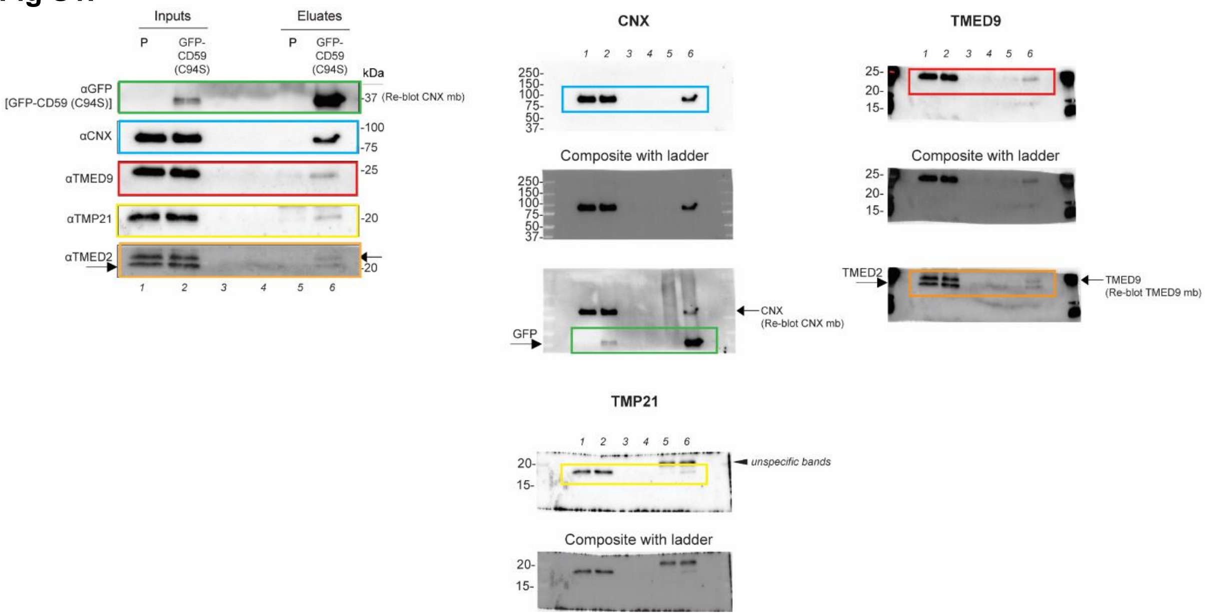

Ronzier and Satpute-Krishnan
Supplementary Blot Raw Data

Figure S3

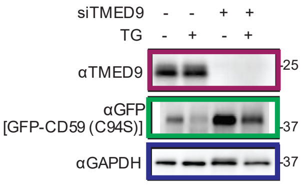

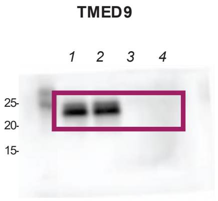

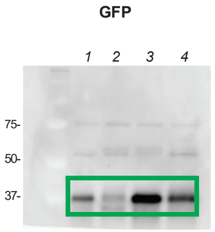

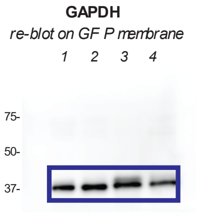

Ronzier and Satpute-Krishnan
Supplementary Blot Raw Data

Figure S5

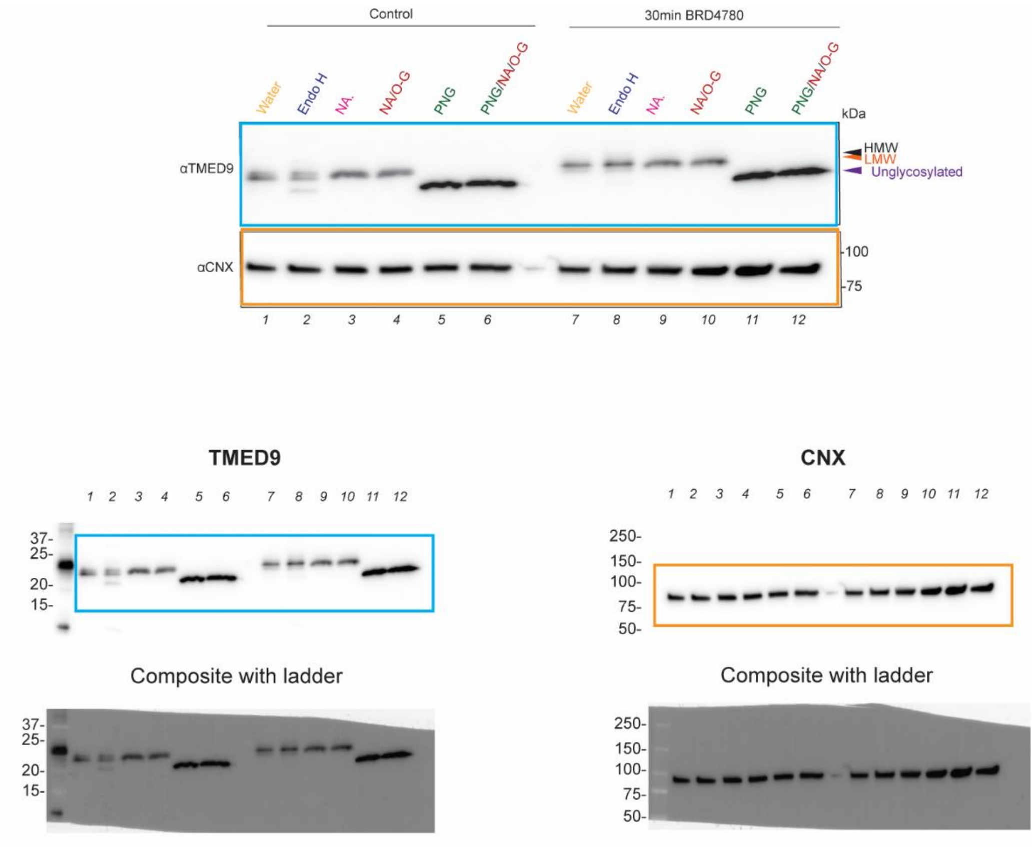

Ronzier and Satpute-Krishnan
Supplementary Blot Raw Data

Figure S7

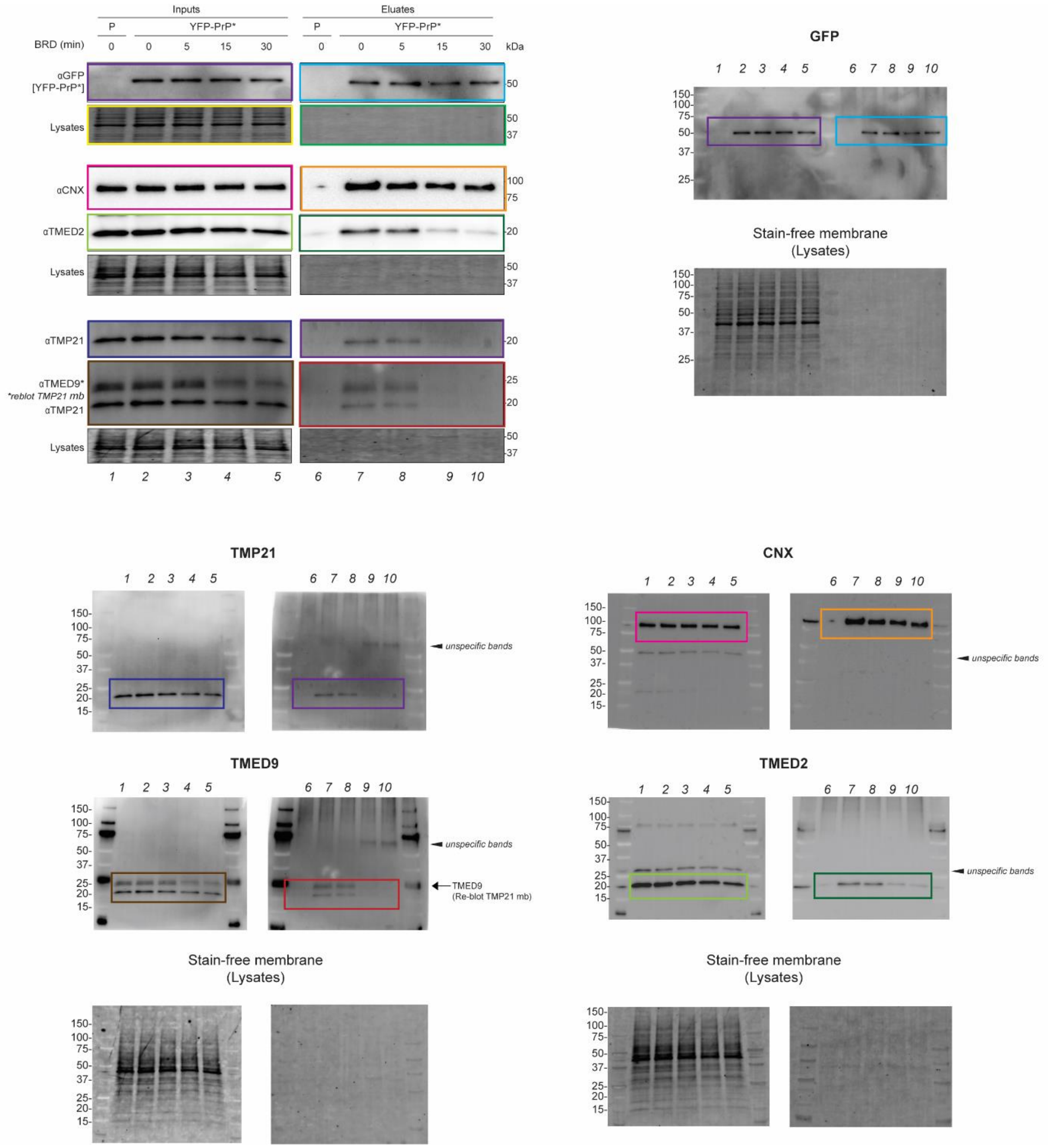

Supplement: S1 Raw Images — (PDF) [file pbio.3003084.s013.pdf]
